# Wet-Spinning Assembly of Continuous, Highly Stable Hyaluronic/Multiwalled Carbon Nanotube Hybrid Microfibers

**DOI:** 10.3390/polym11050867

**Published:** 2019-05-13

**Authors:** Ting Zheng, Nuo Xu, Qi Kan, Hongbin Li, Chunrui Lu, Peng Zhang, Xiaodan Li, Dongxing Zhang, Xiaodong Wang

**Affiliations:** 1 School of Materials Science and Engineering, Harbin Institute of Technology, Harbin 150001, China; zthappy1127@gmail.com (T.Z.); xunuo_hit@163.com (N.X.); luchunrui06@126.com (C.L.); ZP1249558527@126.com (P.Z.); 2 College of Materials Science and Chemical Engineering, Harbin Engineering University, Harbin 150001, China; 3 AVIC Aero Polytechnology Establishment, Beijing 100028, China; 15945698715@163.com; 4 College of Light Industry and Textile, Qiqihar University, Qiqihar 161000, China; lhb987258@163.com; 5 School of Chemistry and Chemical Engineering, Jinggangshan University, Ji’an 343009, China

**Keywords:** multiwalled carbon nanotube, hyaluronic acid, microfibers, wet-spinning, microstructures, tensile properties

## Abstract

Effective multiwalled carbon nanotube (MWCNT) fiber manufacturing methods have received a substantial amount of attention due to the low cost and excellent properties of MWCNTs. Here, we fabricated hybrid microfibers composed of hyaluronic acid (HA) and multiwalled carbon nanotubes (MWCNTs) by a wet-spinning method. HA acts as a biosurfactant and an ionic crosslinker, which improves the dispersion of MWCNTs and helps MWCNT to assemble into microfibers. The effects of HA concentration, dispersion time, injection speed, and MWCNT concentration on the formation, mechanical behavior, and conductivity of the HA/MWCNT hybrid microfibers were comprehensively investigated through SEM, UV-Vis spectroscopy, tensile testing, and conductivity testing. The obtained HA/MWCNT hybrid microfibers presented excellent tensile properties in regard to Young’s modulus (9.04 ± 1.13 GPa) and tensile strength (130.25 ± 10.78 MPa), and excellent flexibility and stability due to the superior mechanical and electrical properties of MWCNTs. This work presents an effective and easy-to-handle preparation method for high-performance MWCNT hybrid microfibers assembly, and the obtained HA/MWCNT hybrid microfibers have promising applications in the fields of energy storage, sensors, micro devices, intelligent materials, and high-performance fiber-reinforced composites.

## 1. Introduction

Carbon nanotubes (CNTs) are very promising materials for electronic and energy storage devices, sensors, biosensors, composites, and transparent conducting films, etc., due to their high specific surface areas, excellent mechanical properties, and good electrical conductivities [1,2,3]. In recent years, individual CNTs have often been assembled into macro/micro structures, such as CNT fibers, CNT films, and CNT arrays, to improve their application [4,5,6]. Among these CNT products, CNT fibers are microsized materials with suitable electrical conductivities, stable electrochemical properties, significant specific moduli, and specific strengths [7,8]. Furthermore, CNT fibers are very flexible and can easily be prepared in different shapes to meet the special requirements of an application, such as woven fabric or artificial muscle bundles [9,10]. For instance, Peining Chen et al. developed actuating fibers that can contract and rotate in response to solvents and vapors [11]. Huhu Cheng et al. wove CNT/G hybrid fibers into textile electrodes for the construction of flexible supercapacitors [12]. Recently, several approaches have been applied to assemble macroscopic fibers of either pure CNTs or CNT-polymer composites. These methods can be summarized as the direct growth of the CNT fibers, dry spinning from a nanotube mat, and coagulation spinning methods [13,14,15,16]. However, it cannot be ignored that the applications of CNT fibers prepared by the first two methods are limited by the high costs of the super-aligned carbon nanotube arrays, strict conditions, complex operational procedures, and low quantities from production [17]. In comparison, the latter process, reminiscent of the so-called wet-spinning method, is particularly simple and potentially scalable for large-scale production. In this method, homogeneous CNTs will be injected into a rotating coagulation medium by a syringe to form a fiber shape. The most common approach for getting stabilized and uniform CNTs dispersion involves the use of surfactants, which can be adsorbed on the out surface of each carbon nanotube to overcome the attractions of van der Waals. The common surfactants are sodium dodecyl sulfate (SDS), sodium dodecyl benzene sulfonate (SDBS), lithium dodecyl sulfate (LDS), and triton X-100. It is worth noting that biomolecules such as single-stranded DNA [10] and chitosan (CHI) [18] have also showed good dispersion property for CNTs in recent literature [19,20]. The typical coagulation medium is polyvinylalcohol (PVA), and other polymer coagulation mediums such as polyethylene-imine (PEI) and polylactic acid (PLA) are also reported. The diameter of CNT fibers varies from several microns to 100 microns, which is mainly affected by the processing conditions, such as the diameter of the syringe, the flow rate of the injection solution, and the condition of the polymer coagulant. Thus, wet-spinning preparation technology of CNT fibers can use any disordered carbon nanotube powder or array as raw material to process into fibers. The raw materials are low cost, equipment is simple, and the operation is easy. However, very few studies have been made on macroscopic finely assembled MWCNT fibers with a controllable, uniform, and large-scale synthetic method that can be used for potential applications until now. Because this method still has many shortcomings, deficiencies need to be further solved. For example, more surfactants need to be added when the concentration of carbon nanotubes is high, large amounts of surfactants can easily form micelles and influence the structure and properties of CNT fibers. Moreover, a large number of studies have found that the obtained performances of these CNT fibers are far less than those of single CNT [21,22]. The main reasons for this are the uneven dispersion of the CNTs and the large number defects in the CNT fibers that will lead to the concentration of stress during the tensile process. The stress cannot be transferred effectively between CNTs, which affects the strength of the CNT fibers. In addition, the defects in the fibers increase the contact resistance between CNTs and affect the electronic transmission in the fibers.

To address the problems above, hyaluronic acid, a biomolecule, was utilized as a surfactant to obtain a stable and homogeneous dispersion of MWCNTs. In contrast with molecular surfactants (SDS or LDS), proportionally equivalent or lower amounts of biomolecules to CNTs were employed to generate a homogenous dispersion, whereby ratios of molecular surfactants of at least 2:1, and in some cases 3:1, were required [18,23]. Secondly, a calcium chloride (CaCl_2_) solution in ethanol was chosen to replace cement polymer coagulant medium to reduce the effect of the polymer on the electrical conductivity of the MWCNT fibers. The fiber structure was obtained through the formation of calcium bridges between the D-glucuronic acid residues on adjacent chains of HA. The effects of different spinning parameters and different concentrations of MWCNTs on the formation, and electrical and mechanical properties of HA/MWCNT hybrid microfibers were discussed, the optimization of the spinning process was completed, and the structure and flexibility of the obtained HA/MWCNT hybrid microfibers were investigated. Based on the results, our wet-spinning system is an effective and easy-to-handle method to assemble MWCNT hybrid microfibers with high strength, conductivity, stability, and flexibility. The obtained high performance HA/MWCNT hybrid microfibers have promising applications in the fields of energy storage, sensors, micro devices, intelligent materials, and high-performance fiber-reinforced composites.

## 2. Materials and Methods 

### 2.1. Materials

MWCNTs purchased from TimesNano Company (Chengdu, China) were used in this study. HA with a molecular weight (MW) of 41–65 kDa, was purchased from Lifecore Biomedical LLC (Chaska, MN, USA). CaCl_2_ was purchased from Sigma–Aldrich (St. Louis, MO, USA).

### 2.2. Fabrication of the HA/MWCNT Hybrid Microfibers

The HA/MWCNT hybrid microfibers were fabricated by the wet-spinning method as below. Firstly, an HA solution was prepared by dissolving HA in deionized (DI) water at room temperature. Then, MWCNTs were added and dispersed in the HA solution using a probe sonicator (YM-1000Y, Shanghai Yuming Instrument Ltd., Shanghai, China) to form the spinning solution. The spinning solution was injected into a rotating coagulation bath (20 rpm) using a syringe pump (LSP02-1B, Dichuang Electronic Technology Ltd., Baoding, China) with a detachable needle (diameter of 0.2 mm) to control the flow rate of injection. The coagulation bath was filled with 5 wt% CaCl_2_ in 70% ethanol. Next, the wet-spun hybrid microfibers were removed from the coagulation bath and rinsed using ethanol and DI water to remove residual coagulating agents. Finally, the prepared hybrid microfibers were dried in air under tension to obtain several meters of the hybrid microfibers.

### 2.3. Characterization and Measurements

The morphologies and microstructures of the HA/MWCNT hybrid microfibers were examined by a scanning electron microscope (SEM) (Merlin Compact, Carl Zeiss AG, Jena, Germany) and 3D digital microscopy (DSX-CB, Olympus Ltd., Tokyo, Japan). UV–Vis scanning spectrophotometry was used to study the dispersion of CNTs suspension. Fourier transform infrared spectroscopy (FTIR, Spectrum One, PerkinElmer, Boston, MA, USA) measurements were recorded at room temperature to confirm the presence of HA. The thermal properties of the HA/MWCNT hybrid microfibers were investigated by differential scanning calorimetry and thermogravimetry analysis (DSC/DTA-TG, STA449F3, Netzsch, Selb, Germany) in a nitrogen environment. The samples were heated from room temperature to 800 °C with a heating rate of 20 °C/min under a nitrogen flow of 50 mL/min. The percentages of the HA and MWCNTs in the HA/MWCNT hybrid microfibers were quantified by the thermogravimetric analysis (TGA) results. The mechanical properties of the HA/MWCNT hybrid microfibers were determined using a uniaxial tensile tester machine (T150 UTM, Agilent, Santa Clara, CA, USA) with a cell load capacity of 10 N at a 0.5 mm/min rate. The electrical conductivities of the HA/MWCNT hybrid microfibers were measured by the four-point probe method using a SourceMeter (Keithley 2400, Tektronix INC., Beaverton, OR, USA). Silver paste was used at the contact points between the hybrid microfibers and the electrode probes to eliminate the contact resistance.

## 3. Results

### 3.1. Dispersion of the MWCNTs in Different Surfactant Suspensions

**Figure 1:**
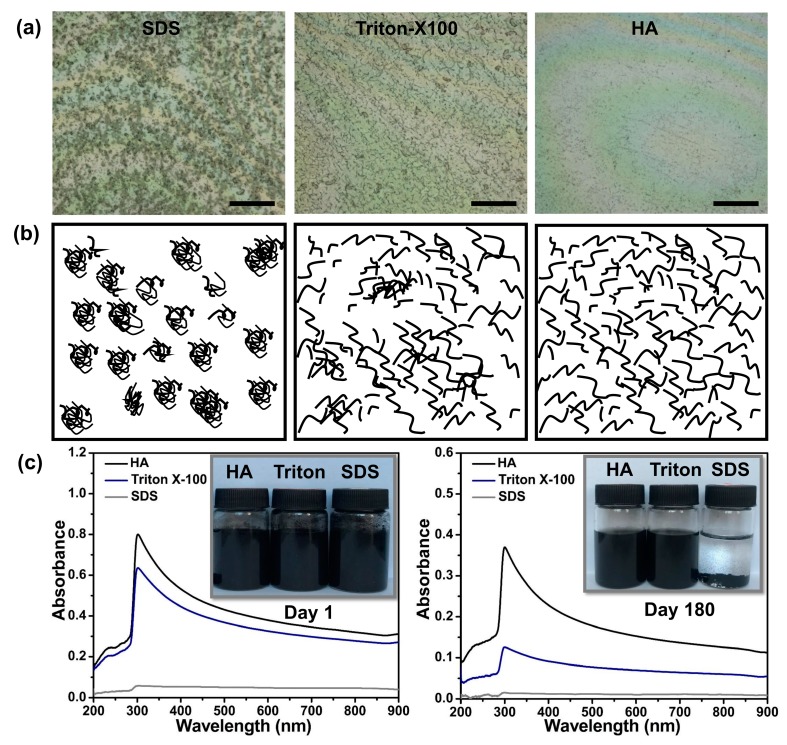
Dispersion of the MWCNTs in different surfactant suspensions. (**a**) Optical images of the MWCNT dispersions (scale bar: 500 μm); (**b**) schematic diagrams of the MWCNT dispersions and (**c**) photos and UV-Vis spectral curves of the MWCNT dispersions.

The dispersion of the CNTs is one of the important primary parameters, which is used as the starting point for further processing into fibers, films, and composites. In general, ionic surfactants (SDS) and nonionic surfactants (TritonX-100) are usually used as dispersion systems for CNT suspensions [24,25]. Therefore, we first compared the bio-surfactant (HA) dispersion stability of the MWCNTs with those of SDS and TritonX-100. The dispersal of MWCNTs in different dispersion systems were studied by 3D digital microscopy and UV–Vis scanning spectrophotometry, as shown in Figure 1. The MWCNT dispersions were too dark to clearly discern the amount of sediment, as shown in the inset of Figure 1c. Thus, the MWCNT dispersions with different surfactants were further observed under a microscope. The results and schematic diagrams of the dispersal states are shown in Figure 1a,b. In the case of a low degree of dispersion by SDS, several agglomerates are observed in Figure 1a. As the degree of dispersion increases, agglomerates become looser, and HA shows the best dispersion behavior. These results were also confirmed by UV–Vis spectroscopy. Figure 1c shows the UV–Vis spectra and photos of the MWCNTs dispersion with different surfactants on day 1 and day 180. The MWCNT dispersions exhibit a characteristic peak at approximately 300 nm, and the absorbance gradually decreases from the UV to near-IR region due to scattering. Increasing the amount of dispersed MWCNTs will result in an increase in the area below the spectral lines that represent the absorbance [26,27]. In addition, HA and TritonX-100 show high absorbance intensities and good dispersion stabilities over a long timeframe. Hence, HA has the highest dispersing power of these three surfactants according to the above experimental results. 

### 3.2. The Morphologies and Microstructures of the HA/MWCNT Hybrid Microfibers

**Figure 2:**
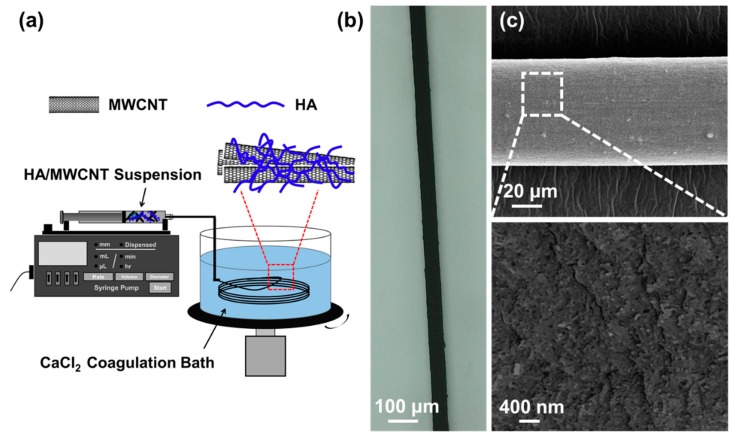
(**a**) A schematic of the experimental design of the wet-spinning method. (**b**) 3D microscopy image of the HA/MWCNT hybrid microfibers drawn from the CaCl_2_ coagulation bath. (**c**) SEM image of the HA/MWCNT hybrid microfibers and an enlarged image of the fibers.

Controlled injections of the HA/MWCNT dispersions into the CaCl_2_ in ethanol coagulating medium afforded a continuous fiber structure due to the formation of calcium bridges between the D-glucuronic acid residues on adjacent chains of HA. A rotating coagulation bath was used to produce a continuous meter-long spinning of the HA/MWCNT hybrid hybrid microfibers; this process is shown in Figure 2a. The long wet HA/MWCNT microfiber was then dried by stretching. Figure 2b shows a microscope photograph of the long fibers collected from the CaCl_2_ solution by wet spinning of a HA/MWCNT dispersion. The fibers were uniformly circular in both the wet and dry states and were swollen and flexible when wet but became brittle and less flexible upon drying. The dry HA/MWCNT hybrid microfibers do not swell when submerged into solution. SEM images were then used to characterize the morphologies of the HA/MWCNT hybrid microfibers. Figure 2c clearly demonstrates that the HA/MWCNT hybrid microfibers are rigid and have a uniformly cylindrical shape. The diameter of the dry HA/MWCNT hybrid microfibers is approximately 70 µm. The enlarged SEM image of the HA/MWCNT microfiber suggests that the microfiber is composed of a number of carbon nanotubes, and the outer surface is very rough, with numerous wrinkles on the surface, which are likely attributable to the roughness of the inner surface of the needle orifice and the shrinkage of the fiber under stretching during drying.

### 3.3. Effects of HA/MWCNT Ratio 

**Figure 3:**
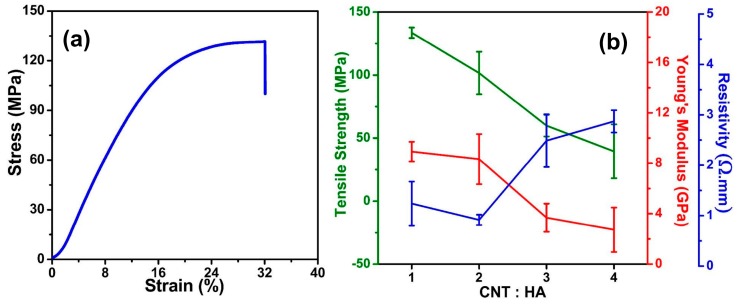
The mechanical and conductivity properties of the HA/MWCNT hybrid microfibers with different ratios of HA to CNTs. (**a**) The stress-strain curve of a HA/MWCNT hybrid microfiber; (**b**) The tensile strengths, Young’s moduli and conductivities of HA/MWCNT hybrid microfibers prepared from different HA:MWCNT ratios.

HA not only influences the dispersion of the MWCNTs but also influences the content ratio of HA to MWCNTs in the hybrid microfibers and further determines the composition and properties of the obtained HA/MWCNT hybrid microfibers. To optimize the amount of HA, the mass ratios of MWCNTs to HA were studied with 1.2% MWCNTs under a 60 mL/h injection speed, and the results are displayed in Figure 3. Figure 3a shows the stress–strain curves of the HA/MWCNT hybrid microfibers during tensile testing, and two stages can be identified from the curve. First, HA/MWCNT hybrid microfibers show a linear relationship between stress and strain, suggesting an initially elastic-like deformation process during stretching, which is reversible. During the elastic-like deformation, the van der Waals forces and the friction between MWCNTs carry little stress. Next, the stress of the HA/MWCNT hybrid microfibers increased nonlinearly with strain before breaking, as fibers were further strained. MWCNTs will bear more of the load, leading to stretching and sliding of the MWCNTs inside the fiber. This is a plastic behavior that is irreversible [18]. Finally, once the tensile stress exceeds the critical value, local stresses cannot be further transferred to neighboring CNTs, and the fibers break. The tensile strengths and Young’s moduli of the HA/MWCNT hybrid microfibers were similar when the mass ratios of MWCNTs to HA were 1:1 and 2:1 and then decreased significantly after reducing the contents of HA according to the results shown in Figure 3b. It is worth noting that the mass ratio of 4:1 produced weak and brittle hybrid microfibers that were difficult to handle during dry processing. This is because the continuous fiber structure was induced by the formation of calcium bridges between D-glucuronic acid residues on adjacent chains of HA, low concentrations of HA will influence the dispersion of the MWCNTs and the formation of the MWCNT fibers. The uneven dispersion of the MWCNTs and the large number of defects in the MWCNT fibers will further lead to the concentration of stress during the tensile process. The stress cannot be transferred effectively between MWCNTs, which affects the strength of the MWCNT fibers. Meanwhile, the defects in the fibers increase the contact resistance between MWCNTs and affect the electronic transmission in the fibers. Therefore, the resistivity of HA/MWCNT hybrid microfibers increased after an initial decline, and the lowest resistivity was 0.92 ± 0.10 Ω∙mm when the mass ratio of MWCNTs to HA was 2:1. The electrons could not be transferred effectively if there were a number of defects in the fibers. Considering the balance between properties, a 2:1 mass ratio of MWCNTs:HA is determined to be the optimal conditions for combining the results of the mechanical and conductivity properties.

### 3.4. Effects of Sonication Duration 

**Figure 4:**
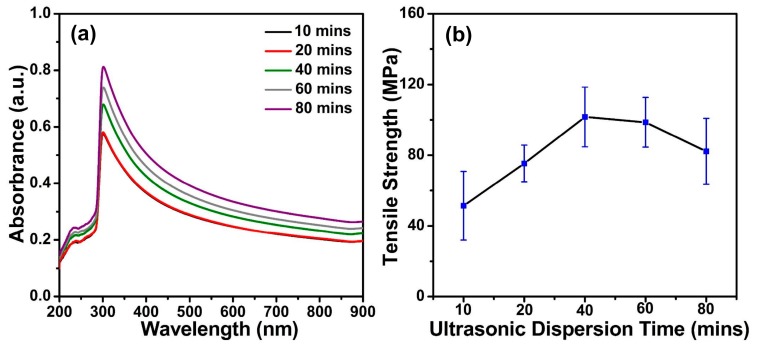
The UV–Vis spectra and tensile strength of the HA/MWCNT hybrid microfibers prepared by HA/MWCNT dispersions with different ultrasonication times. (**a**) UV-Vis spectra; (**b**) Tensile strength results.

Ultrasonication is an external mechanical energy that can help MWCNT bundles overcome the attractive van der Waals forces to disentangle [28]. Therefore, an effective way to disperse CNT solutions is by controlling the sonication time and supplied energy. Additionally, the absorbance of the CNT suspension at a specific wavelength can be related to the degree of debundling of the CNTs in solution [29]; thus, the peak intensity of the obtained UV–Vis spectra is an effective tool to monitor the sonication dispersion process. In this case, the ultrasonic power was set to a fixed value, and HA/MWCNT solutions treated by different ultrasonication times were investigated. Figure 4a illustrates the UV–Vis spectra of HA/MWCNT solutions with different ultrasonication times. The UV–Vis spectra exhibited a characteristic peak at approximately 300 nm, as discussed above. Additionally, the longer the ultrasonication duration, the greater the intensity of the characteristic peak. This means that extending the ultrasonication time can help to well disperse the MWCNTs in the HA solution. However, the ultrasonication process contains two mutually antagonistic effects, one being the deagglomeration of the MWCNTs and the other being the fragmentation of individual CNTs [30]. In other words, individual CNTs will be broken, if the ultrasonication duration is too long. In fact, the dispersion of the MWCNTs is an important factor that can not only affect the length and diameter of the MWCNTs but also further influence the formation and properties of the HA/MWCNT hybrid microfibers. The mechanical performance is one of the crucial parameters of MWCNTs for their practical application. Therefore, the mechanical properties of the HA/MWCNT hybrid microfibers prepared by suspensions with different ultrasonication times were investigated, and the results are displayed in Figure 4b. The tensile strength first increased as the ultrasonication times were increased due to the efficient dispersion of MWCNTs and then tended to decrease slightly for longer sonication times due to the destruction of the aspect ratio of individual CNTs by the sonication energy. Hence, 40 min was chosen as the optimal ultrasonication time for later experiments by combining the results of the UV–Vis spectra and tensile properties.

### 3.5. Effects of Injection Speed

**Figure 5:**
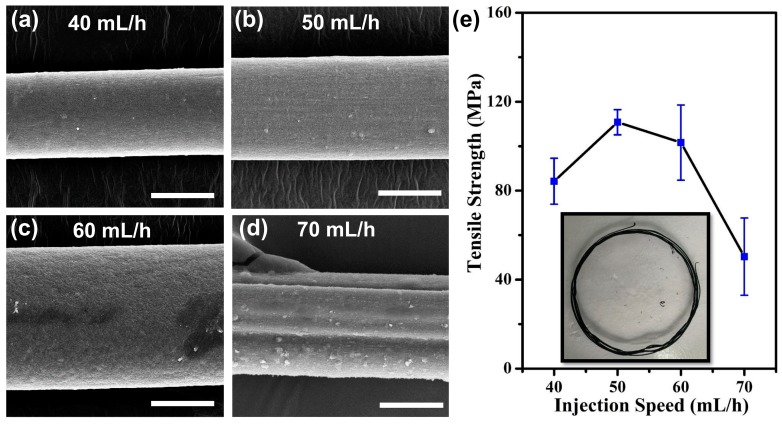
SEM images and tensile strength of the HA/MWCNT hybrid microfibers prepared with different injection speeds (scale bars: 40 μm). (**a**) Injection speed is 40 mL/h; (**b**) Injection speed is 50 mL/h; (**c**) Injection speed is 60 mL/h; (**d**) Injection speed is 70 mL/h; (**e**) Tensile strength results.

After sonication, HA/MWCNT dispersions were injected into a rotating coagulation bath to produce continuous meter-long fibers as required. In a previous report, the ratio of the injection speed to the rotating speed of the rotator acted as an important parameter for the formation and performance of wet-spun CNT fibers [18,31]. In our case, a 1.2% optimal HA/MWCNT suspension was injected into a 20 r/min rotating coagulation bath at different speeds (30 mL/h, 40 mL/h, 50 mL/h, 60 mL/h, 70 mL/h, and 80 mL/h). The HA/MWCNT hybrid microfibers were difficult to handle for subsequent processing and characterization under injection rates of 30 mL/h and 80 mL/h and the graphs of HA/MWCNTs hybrid microfibers prepared with other injection speeds (40 mL/h~70 mL/h) were shown in Figure S1. Only short fibers could be formed and removed from the coagulation bath when a 40 mL/h injection speed was applied. As shown in Figure 5, the diameter of the HA/MWCNT hybrid microfibers prepared by 40 mL/h injection speed was 55.42 ± 5.84 μm, and increased to 65.8 ± 8.28 μm and 88.12 ± 6.72 μm for the microfibers fabricated under 50 mL/h and 60 mL/h injection speed, respectively. Moreover, ribbon-like fibers were obtained and then became hollow and tubular after drying when higher injection rates were used to prepare the samples, as shown in Figure 5d. This morphology is likely caused by the shaking of the needle under faster injection rates. The tensile strength of the HA/MWCNT hybrid microfibers prepared at different injection speeds was also calculated. The results are shown in Figure 5e. The HA/MWCNT hybrid microfibers made with a 50 mL/h injection speed formed well in the coagulation bath (Figure 5b, inset) and possessed the highest mechanical properties of the different samples. As discussed above, the HA/MWCNT hybrid microfibers were easily broken during drying and a low fiber-forming rate was obtained when the injection speed was too low. When the injection speed was too fast, the HA/MWCNT hybrid microfibers became ribbons with poor mechanical properties that tended to curl easily. In this case, an injection speed of 50 mL/h was chosen as the suitable parameter for the subsequent MWCNT fiber preparations.

### 3.6. Effects of MWCNT Concentration

**Figure 6:**
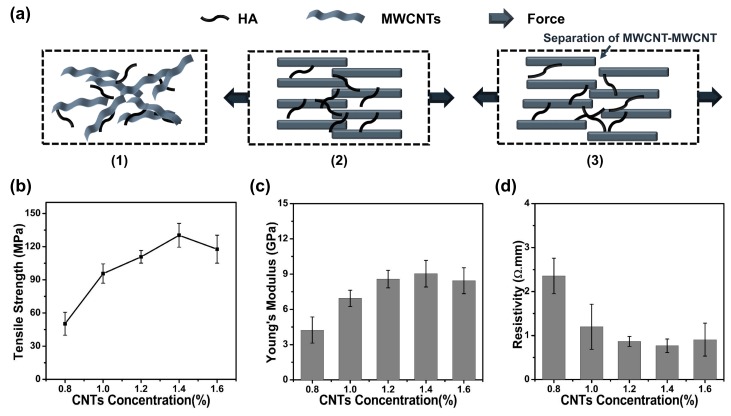
Mechanical properties and resistivities of the HA/MWCNT hybrid microfibers produced with different MWCNT concentrations. (**a**) The fracture mechanism model of the HA/MWCNT hybrid microfibers under tensile stress; (**b**) Tensile strength; (**c**) Young’s Modulus; (**d**) Resistivity.

Figure 6 shows the comparison of the mechanical properties and resistivities of HA/MWCNT hybrid microfibers prepared with different MWCNT concentrations. As expected, the mechanical properties of the HA/MWCNT hybrid microfibers exhibited an increasing tendency with increasing MWCNT concentration due to the interfacial interactions, the efficient transfer of load and energy, reducing concentrated stress and dissipating the energy induced by stretching the fibers [32]. The Young’s modulus of the samples varies from 4.24 ± 1.11 GPa for the 0.8% MWCNT hybrid microfibers to 9.04 ± 1.13 GPa for the 1.4% MWCNT microfiber, while the tensile strength increases from 50.27 ± 10.37 MPa to 130.25 ± 10.78 MPa, respectively, as shown in Figure 6c,d. However, when the MWCNT concentration increased to 1.6%, both the Young’s modulus and tensile strength exhibited a decreasing trend, declining to 8.44 ± 1.10 GPa and 117.70 ± 12.68 MPa, respectively. The fracture mechanism model of the HA/MWCNT hybrid microfibers under tensile stress is shown in Figure 6a. During the tensile test, the stress is first applied to both ends of the microfiber in the axial direction, and subsequently, the fibrous elements endured a pulling force to straighten the MWCNTs. Next, fibers were further strained under force, and the MWCNTs bore a greater load, which lead to the stretching and sliding of the MWCNTs inside the hybrid microfibers. As the force further increased, the stress could no longer be transferred effectively [21]. Hence, MWCNTs in the fibers separated from one another, and the fiber broke. During this process, the interfacial binding and friction force between the MWCNTs may play an important role, and the existence of HA may improve the connections between individual MWCNTs. In all probability, the slight decrease in mechanical properties for high MWCNT concentration samples was caused by the poor distribution and weak interfacial contact between MWCNTs or MWCNT bundles.

The variation in the MWCNT concentrations could also control the electrical properties of the HA/MWCNT hybrid microfibers, which is another important parameter for their future application in fields such as electrical and electrochemical. Accordingly, the resistivities of the HA/MWCNT hybrid microfibers made with different CNT concentrations were investigated, and the results are displayed in Figure 6d. It is expected that the resistivity of the HA/MWCNT hybrid microfibers would decrease with increasing CNT concentrations due to the formation of efficient electrical pathways. However, the resistivity of HA/MWCNT hybrid microfibers with different CNT concentrations shows similar results to those of the mechanical properties. The resistivity decreased from 2.35 ± 0.40 Ω∙mm to 0.77 ± 0.55 Ω∙mm as the CNT concentration was varied from 0.8% to 1.4% and then increased to 0.91 ± 0.37 Ω∙mm when the CNT concentration was further increased. This change can be explained by a number of reasons [5,33,34]. First, some of the MWCNTs are wrapped with a thin coating around a single tube, which may be an impediment to forming a conductive path. Importantly, the higher the concentration of MWCNT hybrid microfibers, the more HA hydrogel is in the fibers. It is generally known that materials made up of nonconducting materials and conducting carbon nanotubes exhibit a significant increase in their conductivities due to higher concentrations being needed to form electrical pathways through the material, i.e., the so-called percolation threshold [16]. For high-concentration HA/MWCNT hybrid microfibers, numerous overlapping carbon nanotubes form a well-connected electrical pathway with increasing concentrations of MWCNTs. However, the resistance of the fibers is controlled by the resistance of the junctions between overlapping MWCNTs. Therefore, it is most likely that HA may cause an increase in the junction resistance of the MWCNT network, which would produce similar results. The combination of the trends observed in the SEM images, mechanical properties and resistivities indicates that HA/MWCNT hybrid microfibers with the optimal properties were prepared by injecting a homogeneous HA/MWCNT dispersion by a 0.2 mm diameter syringe at a rate of 50 mL/h into a CaCl_2_ coagulation bath, which was simultaneously rotated at 20 r/min. The homogeneous HA/MWCNT dispersion with a ratio of 1:2 was ultrasonicated in a water bath using a high-powered sonic tip (500 W, 36% amplitude) under pulse mode (1 s on, 1 s off) for 40 min.

### 3.7. Characterization of the HA/MWCNT Hybrid Microfibers

**Figure 7:**
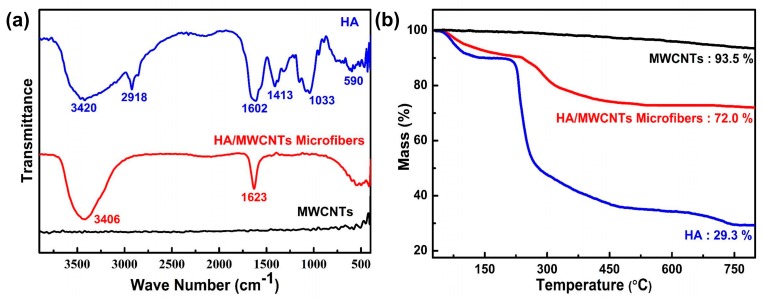
FTIR spectra and TGA curves of HA, MWCNTs, and HA/MWCNT hybrid microfibers. (**a**) FTIR spectra; (**b**) TGA curves.

Further testing was carried out to characterize the functional groups in the fibers by FTIR. The FTIR spectra of the MWCNTs, HA, and HA/MWCNT hybrid microfibers are shown in Figure 7a. The spectrum of the CNTs shows weak absorption bands and could not be detected by FTIR. For the spectrum of the HA/MWCNT hybrid microfibers, the absorption band between 3600 and 2800 cm^−1^ is assigned to the stretching vibrations of O–H and C–H, which are typical characteristics of polysaccharides. The typical absorption peak at 1567 cm^−1^ corresponds to overlapping vibrations of the acetamide and carboxylate groups [35]. These two obvious absorption bands can be utilized to verify the inclusion of HA in the MWCNT hybrid microfibers. However, many of the HA absorption peaks are weak due to the low content of HA in the hybrid microfibers. For instance, the absorption peak at 1033 cm^−1^ is attributed to the skeletal vibrations of the saccharide structure, which involve C–O stretching. The peaks at 1413 cm^−1^ (–C–H bending) and 597 cm^−1^ (N–H vibration) are also present in the spectrum of HA [36]. 

To demonstrate the thermal stability of the HA/MWCNT hybrid microfibers and quantify the MWCNT and HA contents in the hybrid microfibers, TG analysis of the MWCNTs, HA, and HA/MWCNT hybrid microfibers was obtained and the results are shown in Figure 7b. The HA/MWCNT hybrid microfibers followed a similar decomposition trend as that of HA. Namely, before 100 °C, there is a gradual rate of weight loss for both HA and the HA/MWCNT hybrid microfibers, which is primarily attributed to the expulsion of absorbed water. Then, both samples start to rapidly degrade at approximately 210 °C due to the random chain scission of the HA polymer chains, and the maximum rate of weight loss temperature occurs at approximately 230 °C. More importantly, it should be noted that the microfiber peak positions of differential thermal gravity (DTG) move towards higher temperatures compared with those of the HA samples, as shown in Figure S2, suggesting an excellent thermal stability of the HA/MWCNT composite after assembling into hybrid microfibers. This is a consequence of the high thermal stability and low weight loss of the carbon nanotubes, which allows for very efficient heat transport in the samples. Moreover, this enhancement could reveal the strong interactions between the MWCNTs and HA, which may noticeably decrease the segmentation motions of the molecular chain, hence slowing down the decomposition process. Additionally, TGA was also performed to study the quantity of CNTs within the fibers according to the residual weight at 800 °C. The residual mass of MWCNTs, HA, and HA/MWCNTs hybrid microfibers was approximately 93.5%, 29.3% and 72.0%, respectively. Therefore, the exact amounts of HA and MWCNTs in the fibers were 33.5% and 66.5 wt%, respectively, which is consistent with the intended contents of these hybrid microfibers.

### 3.8. The Flexibility and Stability of the HA/MWCNT Hybrid Microfibers

**Figure 8:**
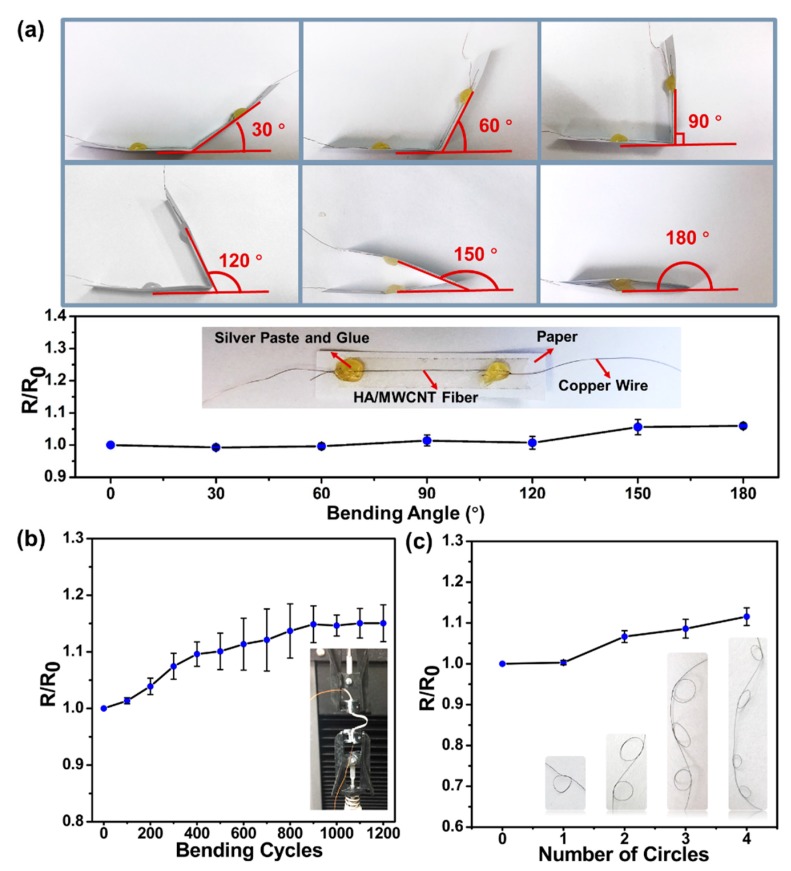
The stability properties of the HA/MWCNT hybrid microfibers. (**a**) Effects of bending angles on the resistance of HA/MWCNT microfibers on a paper substrate; inset: A graphic illustration of the test method for the HA/MWCNT microfibers; (**b**) The resistance stability results of the HA/MWCNT microfibers over 1200 folding-unfolding cycles; inset: A photograph showing the fiber being operated on by the tensile machine; and (**c**) The dependence of resistance on different numbers of tied circles.

In practical applications, fiber-shaped materials should possess excellent flexibility and mechanical stability. Here, the flexibility and stability of the HA/MWCNT hybrid microfibers were characterized by a resistance test, as shown in Figure 8. For convenient and accurate measurements, a fiber was placed on a thin paper substrate, and its two ends were connected to copper wires with silver conducting paste, as shown in the inset of Figure 8a. Figure 8a shows the curve and photographs illustrating the effect of bending angles on the resistance of the HA/MWCNT hybrid microfibers. The results show that the largest relative change of the resistance is 1.00 ± 0.06, revealing a high stability of the HA/MWCNT hybrid microfibers. In addition, the stability of the electrical conductance during cyclical mechanical deformation was examined by folding-unfolding cycles applied to the microfiber using a mechanical tester and the simultaneous measurement of the resistivity (Figure 8b insets). As Figure 8b depicts, more than 1200 cycles were applied to the microfiber, and the resistance was recorded every 100 cycles. The relative change of the resistance was 1.15 ± 0.03 after 1200 cycles, which further confirmed the high stability of the HA/MWCNT hybrid microfibers. Furthermore, the HA/MWCNT hybrid microfibers were twisted into four circles (Figure 8c) to demonstrate their flexibility. As shown in Figure 8c, the relative change in the resistance shows a negligible deviation after twisting into two circles and increases to 1.11 ± 0.02 after being tied in four circles, confirming the flexibility of these fibers, which could be used in micromachine applications. The conductivity stability of MWCNT hybrid microfibers depends not only on electrical properties but also on the mechanical properties of the fibers. In our case, HA provides good mechanical support, while the MWCNTs provide a more stable conductive path. Overall, the HA/MWCNT hybrid microfibers exhibit stabilized electrical conductivity and excellent mechanical properties and could be used in microsized materials, conductive materials, electrode materials, intelligent materials and high-performance fiber reinforced composite materials applications

## 4. Conclusions

In conclusion, continuous, conducting, high strength, and flexible HA/MWCNT hybrid microfibers have been successfully produced by a wet-spinning method using HA as a surfactant and ion-conducting binder in the spinning solution. The effects of HA concentration, dispersion sonication time, injection speed, and MWCNT concentration on the formation, conductivity, and mechanical behavior of the HA/MWCNT hybrid microfibers were comprehensively investigated. An effective and easy-to-handle manufacturing method for MWCNT hybrid microfibers is presented, and show that the obtained HA/MWCNT hybrid microfibers with excellent electrical conductivity, mechanical properties, and stable behavior are a promising material for microsized materials, conductive materials, electrode materials, intelligent materials, and high-performance fiber reinforced composite materials.

## Supplementary Materials

The following are available online at https://www.mdpi.com/2073-4360/11/5/867/s1, Figure S1: The graphs of HA/MWCNTs microfibers prepared with different injection speed, Figure S2: DTG curves of HA and HA/MWCNTs microfibers.
