# Supplementary material for: Wet-Spinning Assembly of Continuous, Highly Stable Hyaluronic/Multiwalled Carbon Nanotube Hybrid Microfibers
Source: Polymers (Basel). 2019 May 13;11(5):867. doi: 10.3390/polym11050867 (PMC6571791; doi:10.3390/polym11050867)
Supplement: Supplementary file 1 [file polymers-11-00867-s001.pdf]

Supplementary Materials

Wet-Spinning Assembly of Continuous, Highly Stable Hyaluronic/Multiwalled Carbon Nanotube Hybrid Microfibers

Ting Zheng, Nuo Xu, Qi Kan, Hongbin Li, Chunrui Lu, Peng Zhang, Xiaodan Li, Dongxing Zhang and Xiaodong Wang

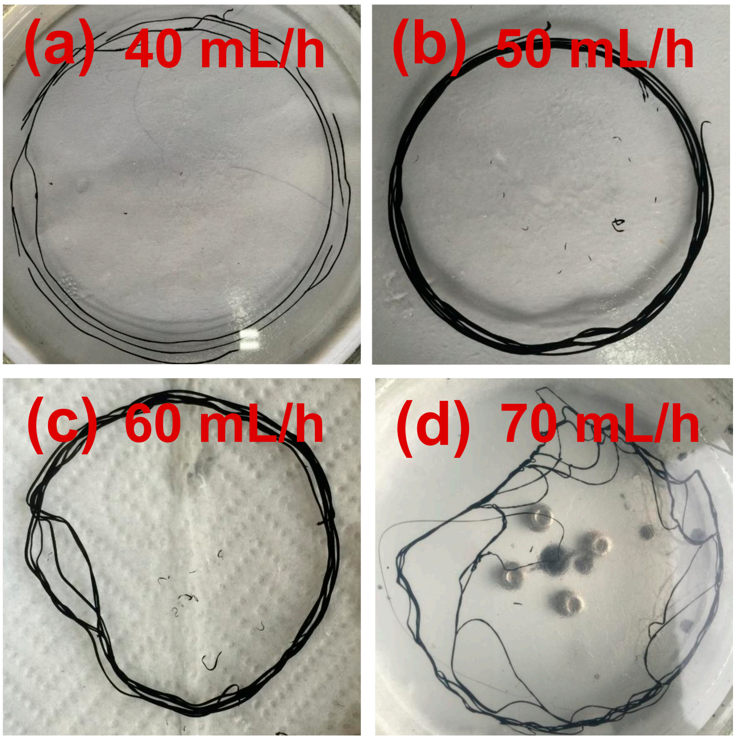

Figure S1. The graphs of HA/MWCNTs microfibers prepared with different injection speed.

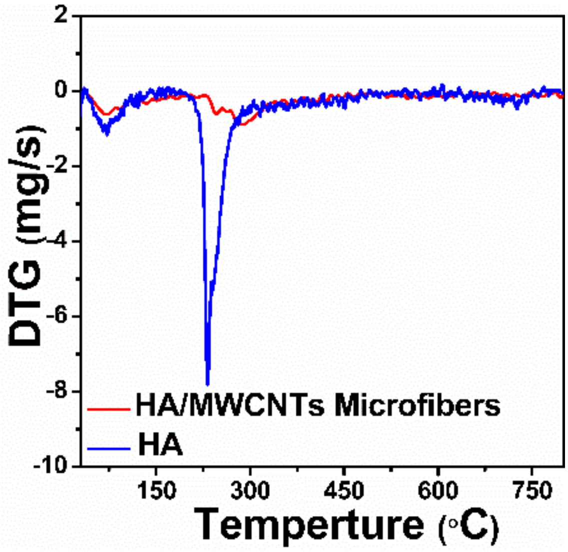

Figure S2. DTG curves of HA and HA/MWCNTs microfibers.
